# Irisin: A Promising Target for Ischemia-Reperfusion Injury Therapy

**DOI:** 10.1155/2021/5391706

**Published:** 2021-10-29

**Authors:** Yani Wang, Huibin Liu, Na Sun, Jing Li, Xiang Peng, Ying Jia, Jason Karch, Bo Yu, Xander H. T. Wehrens, Jinwei Tian

**Affiliations:** ^1^Department of Cardiology, The Second Affiliated Hospital of Harbin Medical University, Harbin 150086, China; ^2^The Key Laboratory of Myocardial Ischemia, Harbin Medical University, Ministry of Education, Harbin 150086, China; ^3^Department of Clinical Pharmacy, The Second Affiliated Hospital of Harbin Medical University, Harbin 150086, China; ^4^Cardiovascular Research Institute, Department of Molecular Physiology & Biophysics, Baylor College of Medicine, Houston, TX 77030, USA; ^5^Cardiovascular Research Institute, Departments of Molecular Physiology & Biophysics, Medicine, Neuroscience, Pediatrics, And Center for Space Medicine, Baylor College of Medicine, Houston, TX 77030, USA

## Abstract

Ischemia-reperfusion injury (IRI) is defined as the total combined damage that occurs during a period of ischemia and following the recovery of blood flow. Oxidative stress, mitochondrial dysfunction, and an inflammatory response are factors contributing to IRI-related damage that can each result in cell death. Irisin is a polypeptide that is proteolytically cleaved from the extracellular domain of fibronectin type III domain-containing protein 5 (FNDC5). Irisin acts as a myokine that potentially mediates beneficial effects of exercise by reducing oxidative stress, improving mitochondrial fitness, and suppressing inflammation. The existing literature also suggests a possible link between irisin and IRI, involving mechanisms similar to those associated with exercise. This article will review the pathogenesis of IRI and the potential benefits and current limitations of irisin as a therapeutic strategy for IRI, while highlighting the mechanistic correlations between irisin and IRI.

## 1. Introduction

Whereas improvements in pharmacological treatments (e.g., thrombolytic drugs) and interventional therapies (e.g., percutaneous transluminal coronary angioplasty) can recover blood perfusion in ischemic heart tissue, the recovery of blood flow (known as reperfusion) can promote metabolic dysfunction associated with cell death. Ischemia-reperfusion injury (IRI) occurs when the blood supply is restored after a temporary loss of oxygen (O_2_) supply (known as ischemia) [1]. As a common pathological phenomenon, IRI occurs in the context of many pathological conditions including acute coronary syndrome, hypovolemic shock, ischemic stroke, and sickle cell disease (SCD). IRI associated with a significant amount of tissue or organ damage can also impact remote organs that were not ischemic during the initial injury [2]. It is currently well established that IRI involves multiple cellular mechanisms, including oxidative stress, mitochondrial dysfunction, and an inflammatory response. While many mechanisms contribute to the pathogenesis of IRI, we will focus on these 3 major pathways and then highlight the specific roles played by irisin, a polypeptide that is proteolytically cleaved from the extracellular domain of fibronectin type III domain-containing protein 5 (FNDC5). Irisin acts as a myokine that is hypothesized to be protective against IRI as it reduces oxidative stress, improves mitochondrial dysfunction, and suppresses inflammation.

## 2. Mechanistic Insights into the Pathogenesis of IRI

### 2.1. Oxidative Stress in IRI

**Figure 1:**
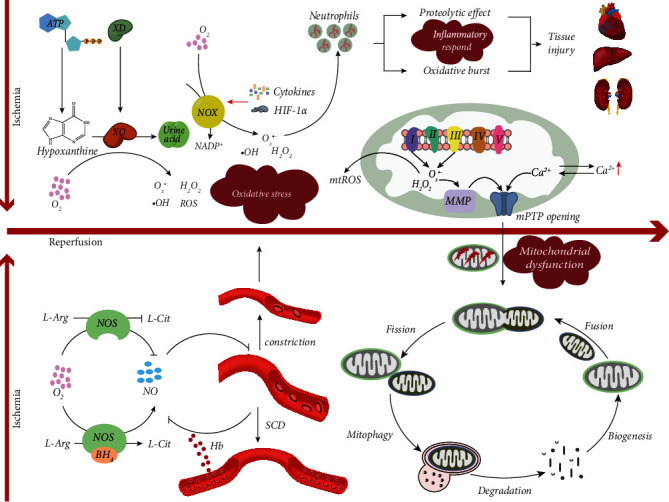
The pathogenesis of ischemia-reperfusion injury on oxidative stress, mitochondrial dysfunction, and the inflammatory response. (1) Oxidative stress occurs and deteriorates with the progression of IRI. In the xanthine oxidase system, XD translates into XO during ischemia; meanwhile, ATP degrades to the XO substrate hypoxanthine, both of which accumulate greatly in ischemic tissues. During reperfusion, the final electron acceptor O_2_ pours into ischemic tissues, causing the transformation from hypoxanthine into uric acid, and the release of superoxide O_2_^•-^, ^•^OH, H_2_O_2_, and ROS. In the NADPH oxidase system, NOX is activated by both activated HIF-1*α* during ischemia and increased cytokines during reperfusion, promoting NADPH translation into NADP^+^ and further inducing massive production of O_2_^•-^, H_2_O_2_, and ^•^OH. In the NOS system, NOS catalyzes O_2_ and L-Arg into L-Cit and NO using cofactor BH_4_, while the content of BH_4_ decreases during IRI to further induce the uncoupling of NOS and the decline of NO. NO has an effect on inhibiting vessel constriction, which may be scavenged during the period of hemolytic anemia and microvascular vasoocclusion in SCD. (2) Mitochondrial dysfunction occurs in IRI. Complex I and complex III contained in the electron transport chain release O_2_^•-^ and H_2_O_2_ and produce mtROS subsequently during IRI. IRI-related mitochondrial oxidative stress and calcium overload trigger MMP collapse and mPTP opening, causing mitochondrial dysfunction. As part of a mitochondrial quality control response, damaged mitochondria may undergo fission, mitophagy, degradation, biogenesis, and fusion to initiate recovery. (3) An inflammatory response occurs during IRI, including neutrophil infiltration, proteolytic effects, and oxidative bursts, which mediate injury in several tissues. ATP: adenosine triphosphate; BH_4_: tetrahydrobiopterin; HIF-1*α*: hypoxia-inducible factor-1*α*; H_2_O_2_: hydrogen peroxide; IRI: ischemia-reperfusion injury; L-Arg: L-arginine; L-Cit: L-citrulline; MMP: mitochondrial membrane potential; mPTP: mitochondrial permeability transition pore; mtROS: mitochondrial ROS; NADPH: nicotinamide adenine dinucleotide phosphate; NO: nitric oxide; NOS: nitric oxide synthase; NOX: NADPH oxidase; O_2_^•-^: superoxide; ^•^OH, hydroxyl radical; ROS: reactive oxygen; SCD: sickle cell disease; XD: xanthine dehydrogenase; XO: xanthine oxidase.

In 1985, “oxidative stress” was first introduced by Dr. Helmut Sies as a concept in redox biology and medicine. This term was defined as “An imbalance between oxidants and antioxidants in favor of the oxidants, leading to a disruption of redox signaling and control and/or molecular damage” [3]. Oxidative stress can be divided into enzymic and nonenzymic stress depending on its source, both of which occur as part of the IRI response. Enzymic oxidative stress is generated by the xanthine oxidase (XO) system, the nicotinamide adenine dinucleotide phosphate (NADPH) oxidase (NOX) system, and the uncoupled nitric oxide synthase (NOS) system, among others. Nonenzymic oxidative stress primarily consists of hemoglobin [4]. Oxidative stress is a vital driving force contributing to the acceleration of various pathological cellular processes during IRI (see Figure 1).

#### 2.1.1. Enzymic Systems Involving Oxidative Stress

As a significant component of the XO system, XO and its precursor xanthine dehydrogenase (XD) account for 10% and 90% with existent forms *in vivo*, respectively [5]. Dysfunction of membrane channels induced by the lack of adenosine triphosphate (ATP) leads to abnormal calcium influx during ischemia, which activates calcium-dependent proteases and further promotes XD conversion to XO. Meanwhile, the declining partial oxygen pressure results in further ATP degradation, which causes accumulation of hypoxanthine as the substrate for XO. Following restoration of the blood flow during reperfusion, O_2_ floods into the ischemic area. As the final electron acceptor, O_2_ promotes the transformation of hypoxanthine into uric acid though XO. During this catalysis, vast quantities of superoxide (O_2_^•-^), hydroxyl radical (^•^OH), and hydrogen peroxide (H_2_O_2_)—all types of reactive oxygen (ROS)—are generated and contribute to oxidative stress [6, 7].

In regard to the NOX system, NOX spans two heme moieties, and the cytoplasmic carboxy terminus of NOX has binding domains for NADPH and flavin adenine dinucleotide (FAD) [8]. In this system, O_2_ is the final electron acceptor via the heme groups, FAD and NADPH [9, 10]. During electron transfer, NOX produces large amounts of O_2_^•-^, which leads to spontaneous transformation of O_2_^•-^ into H_2_O_2_*in vivo*, the latter having a direct cytotoxic effect [11, 12]. Additionally, H_2_O_2_ causes cytotoxicity indirectly when metal ions (e.g., ferrous) exist, leading to the production of the highly reactive molecule ^•^OH via the Fenton reaction [13]. During ischemia, activated hypoxia-inducible factor-1*α* (HIF-1*α*) activates NOX, which further induces vast production of O_2_^•-^, H_2_O_2_, and ^•^OH to trigger oxidative stress. In turn, oxidative stress also increases the expression of HIF-1*α* [4]. Moreover, cytokines and other bioactivator molecules are released in large quantities and also activate NOX, thereby further contributing to oxidative stress [2, 14, 15].

NOS is one of dominant oxidative stress factors in cardiovascular and metabolic diseases [16]. Using cofactor tetrahydrobiopterin (BH_4_), NOS catalyzes O_2_ and L-arginine (L-Arg) into L-citrulline (L-Cit) and nitric oxide (NO). NO has vasodilative and anti-inflammatory effects [17]. During IRI, oxidative stress or catabolism results in a decreased BH_4_ content, which induces uncoupling of NOS. As a result, the production of O_2_^•-^ increases. While the generation of NO decreases, leading to increased oxidative stress [4].

#### 2.1.2. Nonenzymic System Involving Oxidative Stress

The pathogenesis of SCD involves mutant *β*-globin chains initiating the polymerization of hemoglobin S and sickling of erythrocytes [18]. Notably, erythrocyte abnormalities in SCD mainly manifest themselves periodically as hemolytic anemia and microvascular occlusion, causing end-organ IRI. Sickle red blood cells (sRBCs) are prone to damage because of their deformation and osmotic fragility. Damaged sRBCs adhere to endothelial cells, resulting in adhesion-mediated vasoocclusion and impaired blood flow followed by accumulation of oxygen free radical and ROS [18, 19]. During the hemolytic phase, the release of free hemoglobin derived from plasma may scavenge NO, and inhibit NO-mediated provasodilative and antioxidative stress effects. Hence, nonenzymic oxidative stress participates in the pathogenesis of IRI and in end-organ damage [18, 19].

### 2.2. Mitochondrial Dysfunction in IRI

Mitochondria dysfunction is a pivotal mediator of IRI [20]. There are several associations between IRI and mitochondrial dysfunction, including electron transport chain- (ETC-) mediated ROS generation and ROS-induced mitochondrial permeability transition pore (mPTP) opening, which all contribute to the induction of the mitochondrial quality control pathway (see Figure 1).

#### 2.2.1. Mitochondrial Oxidative Stress and mPTP Opening

Mitochondria are a major source of ROS generation, in which complex I and complex III, the principal components of the ETC, are of the main contributors to mitochondrial ROS (mtROS) production. Complex I interacts with its metabolic substrates, releasing 2 electrons to the coenzyme Q (CoQ) reduction site by cofactors flavin mononucleotide (FMN), followed by reduction of CoQ to CoQH_2_ and the simultaneous release of 2 electrons. In the mitochondrial ECT, both the FMN domain and the CoQ binding site are sites of superoxide production [21]. In the highly reduced state of the CoQ pool, electrons from CoQH_2_ are forced to return to complex I, a process known as reverse electron transport (RET) [22]. The maximum production of superoxide in RET is much faster than the maximum rate of ETC. Therefore, complex I-related RET is deemed to be the main source of superoxide production during IRI [23]. Complex III participates in ubiquitin oxidation and cytochrome c reduction in the Q cycle, which produces vast amounts of superoxide by mediating the reaction of ubisemiquinone bound to the CoQ site and O_2_ [24]. The superoxide in both of these sources convert into H_2_O_2_ during IRI, which in turn causes mtROS accumulation in large quantities and contributes to mitochondrial dysfunction [25].

Continuous ischemia may trigger cell death and tissue injury depending on the duration of the ischemic period. However, in some cases, the majority of damage actually occurs during the reperfusion phase [23]. The initial few minutes of reperfusion are crucial for the severity of IRI: soon after the start of reperfusion there is a burst of mtROS generation [20, 23]. Ischemia-induced damage alters the metabolic supply and oxygen utilization, leading to the formation of intracellular acidification, triggering intracellular and mitochondrial calcium overload, and mPTP opening subsequently [20, 26, 27]. During reperfusion, the large increase in oxidative stress further sensitizes the mPTP sensitivity to calcium. Thus, the majority of the mPTP opening occurs upon reperfusion [25]. Following mPTP opening, water and solutes will flow freely into the mitochondrial matrix, leading to mitochondrial swelling, loss of the mitochondrial membrane potential, and release of intermembrane space proteins (e.g., cytochrome c and apoptosis-inducing factor). Eventually, caspase-dependent or caspase-independent cell death will occur depending on the overall severity of mitochondrial dysfunction that has occurred within the individual myocyte [28, 29].

#### 2.2.2. Response of Mitochondrial Quality Control

Mitochondrial quality control is a dynamic process that includes fission, fusion, mitophagy, and biogenesis. During oxidative stress, dynamin-related protein 1 (Drp1) translocates to damaged mitochondrial exterior membranes via actin and microtubule-mediated transport mechanisms, and with Drp1 receptors on the outer mitochondrial membrane including mitochondrial fission protein 1 (Fis1) [30–32]. Following the interaction of Drp1 with its receptors, mitochondria appear to constrict and dissociate leading to mitochondrial fission [33, 34]. Moreover, optic atrophy 1 (Opa1) mediates the fusion between the damaged mitochondria and the outer mitochondrial membranes of normal mitochondria in an apparent effort to rescue the abnormal mitochondria by a process known as mitochondrial fusion [35, 36]. In most cases, mitophagy occurs following fission and is degraded by mitophagic proteins to maintain overall mitochondrial integrity. The degraded components will be reused for supplying proteins and lipids to potentially promote the growth of new mitochondria, a process known as mitochondrial biogenesis [37]. Mitophagy and mitochondrial biogenesis are two processes that contribute to the maintenance of mitochondrial content and metabolism.

Zhou et al. [38] found that an ischemia-related cascade can contribute to the triggering of fatal mitochondrial fission in endothelial cells. During reperfusion, Drp1 translocates to the mitochondrial exterior membrane and mediates mitochondrial fission [39, 40]. Additionally, the function of Opa1 is interrupted in a Bax- and Bak-dependent manner, hindering mitochondrial membrane fusion in cardiomyocytes [41, 42]. Together, these changes in mitochondrial dynamics are closely related to heart IRI. Alternatively, Parkin mediates the ubiquitination of Drp1, which reduces mitochondrial fission, as part of a mitochondrial quality control system that is protective against IRI [43]. Indeed, moderate mitophagy increases the survival advantage of cardiomyocytes following IRI; however, overactivation of mitophagy can be detrimental for the cell [44]. There is evidence suggesting that autophagy overactivation induces cortical neural cell injury following cerebral IRI [45]. Likewise, damage-derived overactivation of autophagy occurs in intestinal and myocardial IRI [46, 47]. Therefore, the aforementioned molecules involved in mitochondrial dynamics and quality control are highly relevant to IRI.

### 2.3. Inflammatory Response in IRI

Metabolic disturbance-induced inflammatory responses promote injury of cells and tissues during ischemia and hypoxia, while ischemia-related metabolic disturbances further translate into an excess innate immune response causing deterioration of injury and organ failure following reperfusion (see Figure 1).

#### 2.3.1. Initial Activation of Inflammatory Response

Neutrophils are regarded as major participants in cellular injury events caused by IRI [48, 49]. The formation of a directional chemotactic gradient and the involvement of chemokines mediate neutrophil adherence, exosmosis, and directional migration [50, 51]. Liver injury after ischemia and reperfusion is gradually induced by neutrophil activation and adherence within the liver sinusoidal vasculature, neutrophil migration to the liver parenchyma, and the release of granule enzymes [52, 53]. In terms of hepatic IRI, oxidative stress induces injury within cells directly leading to the release of “damage-associated molecular patterns” (DAMPs), promoting production of inflammatory factors by Kupffer cells [50]. As a ligand, DAMPs combine with toll-like receptors which are upregulated on the surface of neutrophils and stimulate the production of chemokines to initiate the sterile inflammation [50, 54]. The initial activation of a proinflammatory response during ischemia contributes to the subsequent injury that takes place during reperfusion [55].

#### 2.3.2. Amplification of Inflammatory Cascades

The amplification of the inflammatory response during reperfusion is primarily related to the release of ROS and neutrophil granule enzymes. Neutrophils gather on and adhere to the surface of hepatocytes and subsequently appear fully degranulated subsequently, resulting in the release of several proteases [50]. The powerful proteolytic effect of elastase induces endothelial cell injury and organ damage [56]. The reaction between myeloperoxidase and H_2_O_2_ produces hypochlorous acid (HOCl) and hypochlorite (OCl^−^), which can contribute to IRI damage [57]. Ischemia-related metabolic disturbance impedes adenosine synthesis, leading to activation of neutrophil phagocytosis and an inflammatory response. During reperfusion, oxygen uptake by neutrophils increases and oxygen radicals accumulate in excess, causing an oxidative damage [58, 59]. Limiting neutrophil adhesion and production of superoxide mediated by the lack of adenosine alleviates IRI in liver and SCD [60–62]. Together, the initial activation of the immune reaction mediated by inflammatory mediators and cells, as well as the amplifying effect of inflammation mediated by the release of neutrophil proteinase and the oxidative stress, exacerbate IRI.

## 3. The Discovery and Characterization of Irisin

### 3.1. The Discovery of Irisin

Exercise has a protective effect on cardiovascular diseases and metabolic disorders [63]. The endocrine function of cardiac muscle and skeletal muscle promotes the synthesis and release of myokines when the muscle contracts, which is mediated by the transcriptional coactivator peroxisome proliferator-activated receptor- (PPAR-) c coactivator-1*α* (PGC1-*α*). In 1998, Puigserver et al. [64] discovered that the expression of PGC1-*α* in brown fat and striated muscle increases remarkably during cold exposure, exerting adaptive thermogenesis by inducing mitochondrial biosynthesis and coactivation of the function of PPAR-*γ* on the UCP1 promoter. In 2012, Bostrom et al. [65] demonstrated that PGC1-*α* causes muscle cells to secrete a molecule that mediates browning of subcutaneous white adipose, which regulates thermogenesis indirectly. Using gene expression arrays, this molecule was identified as FNDC5, a secreted PGC1-*α*-dependent protein [65]. Moreover, this research confirmed that recombinant FNDC5 could induce brown fat-like conversion of subcutaneous white adipose and accelerated energy expenditure. Subsequently, mass spectrometry uncovered an effective splice product of FNDC5 secreted into the circulation. This exercise-related polypeptide consisting of 112 amino acids was named irisin [65]. In 2018, proteomics and mass spectrometry studies revealed that integrin *α*V/*β*5 acts as the functional receptor of irisin, providing potential targets for the mechanistic regulation of irisin [66]. This new exercise-related polypeptide irisin sparked great interest in the scientific community. As a potential connection between exercise and metabolic disorders, irisin opened a window of opportunity for improving existing treatment protocols and novel therapeutic strategies.

### 3.2. The Characterization of Irisin

#### 3.2.1. Cytoprotection of Irisin

The source of irisin has been studied in Sprague-Dawley rats, which revealed that it is produced more in cardiac muscle than in skeletal muscle [67]. The biological effects and mechanisms of action of irisin in cardiac ischemia have been partly identified since its initial discovery [68, 69]. The protective role of irisin is related to its regulation of energy metabolism and fatty acid metabolism, but involves a heterogeneous effect in several cell types. Irisin promotes cardiac progenitor cell-related myocardial repair [70], improves endothelial dysfunction-induced abnormal vasomotor function [71], and affects macrophage-mediated host defense function [72] to realize a beneficial effect in multiple cardiac disorders. Furthermore, irisin is highly conserved across all mammalian species. The similarity of irisin in human and mouse reaches close to 100% [65]. The high level of conservation of irisin may be suggestive of its important roles in protecting against oxidative stress, mitochondrial dysfunction, and inflammation.

IRI triggers multiple programmed cell death pathways including necrosis, apoptosis, and autophagy, in which the first two are classic death patterns seen in IRI-related disorders and autophagy is an adaptive response to such stress states [73, 74]. Necrosis exerts highly immunostimulatory effects, and apoptosis induces autonomic cell death, both of which results in organ dysfunction [1]. Autophagy plays a beneficial role in IRI by inhibiting apoptosis and eliminating damaged mitochondria. The IRI-related cytoprotection of irisin involves antiapoptosis, proautophagy, and anti-inflammatory effects [75, 76]. Apoptosis has been identified as a main pathophysiological mechanism in myocardial IRI, which can be rescued by irisin's induction to autophagy [74, 77]. Similar protective effects occur in hepatic IRI [78]. In the next chapter, we will discuss the mechanisms by which irisin protects against IRI in detail; however, there are some controversies in the field.

#### 3.2.2. Controversies about the Effects of Irisin

A meta-analysis based on 741 studies showed that irisin concentrations in patients with coronary artery disease (CAD) are lower than those in healthy controls [79]. In advanced cases with CAD, the irisin concentrations are lower than those in the control group and milder cases [80]. Therefore, irisin has been proposed as a biomarker for monitoring the severity of CAD in patients [81]. However, the predictive value of irisin levels are paradoxical in cardiovascular diseases. Based on current research, higher irisin concentrations are correlated with an increased risk of acute coronary syndrome and adverse cardiovascular events [82, 83]. In acute heart failure, patients with higher circulating irisin levels had a higher mortality [84]. Interestingly, polymorphisms in the PGC1-*α* gene are related to an increased risk of hypertrophic cardiomyopathy [85]. Meanwhile, one study showed that a single-nucleotide polymorphism in the irisin gene may be a genetic risk factor for myocardial infarction. The rs3480 genotypes and rs726344 genotypes of the irisin gene are associated with hypertension, diabetes, and hyperlipidemia, demonstrating that both may confer an enhanced susceptibility to MI [86].

These seemingly contradictory effects of irisin might be due to variations of several factors, such as the type and intensity of exercise, the detecting methods and testing times, the baseline concentration of glucose, lipids, and inflammatory factors *in vivo*. In addition to variations in experimental design and techniques, irisin might exert both compensatory and reactive changes as part of the disease pathogenesis, which might explain such heterogeneity as well. On the one hand, the irisin abundance appears to be associated with the stage of disease development [82, 87]. Hence, we infer that higher irisin levels in the early stages exert a protective effect to inhibit myocardiocyte apoptosis and disease deterioration, but increase the risk of adverse events in advanced states. On the other hand, the dose-effect relationship of irisin plays a vital role in cardiac diseases [88]. Thus, studies probing the median effective dose and the median toxic dose of irisin are urgently needed in various cardiac diseases. Meanwhile, the clinical transformation of irisin should also take into account the adverse aspect with its higher energy expenditure [67, 89].

## 4. The Mechanistic Correlation between Irisin and IRI

**Figure 2:**
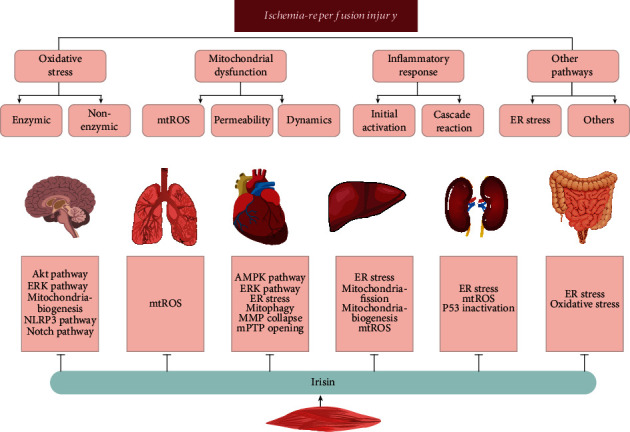
The mechanisms of irisin in ischemia-reperfusion injury in various organs. Ischemia-reperfusion injury occurs in various organs, including the cerebrum, lung, heart, liver, kidney, and intestines. The pathogenesis of ischemia-reperfusion injury includes oxidative stress, mitochondrial dysfunction, an inflammatory response, and other pathways. The roles of irisin in various mechanistic pathways underlying ischemia-reperfusion injury have been studied in different organs. AMPK: adenosine 5′-monophosphate-activated protein kinase; ERK: extracellular-regulated protein kinases; ER stress: endoplasmic reticulum stress; MMP: mitochondrial membrane potential; mPTP: mitochondrial permeability transition pore; mtROS: mitochondrial reactive oxygen; NLRP3: NOD-like receptor family, pyrin domain-containing 3.

Recently, the effects of irisin on antioxidative stress, mitochondrial protection, and anti-inflammatory signaling have been investigated. Strikingly, the main mechanisms and key molecules contributing to IRI correlate with the protective effects of irisin. In the following section, we will focus on the correlation between the effects of irisin and IRI in regard to oxidative stress, mitochondrial dysfunction, and inflammatory response (see Figure 2).

### 4.1. The Role of Irisin in Antioxidative Stress in IRI

Irisin has been shown to play a role in the antioxidative stress response. The balance between oxidase and antioxidase maintained by irisin may delay disease development to some extent. NOX mediates the production of ROS in a rat model of transverse aortic constriction (TAC). The accumulation of ROS has two types of impact, one is the uncoupling of NOS, which causes a decrease in NO production and an increase in ROS concentrations; the second one is activation of XO, which exacerbates oxidative stress and pressure overload-induced cardiac remodeling. Conversely, irisin was shown to inhibit the expression of NOX and XO in cardiac muscle of rats with TAC, and reverse the decline of glutathione peroxidases (GPXs) and superoxide dismutase 1 (SOD1) levels, thereby reducing the level of oxidative stress and damage [90]. Irisin also increases NOS expression and initiates NO generation in the context of obesity or vascular complications of diabetes [91, 92]. Under high-glucose or high-fat conditions, excessive NO interacts with superoxide to produce peroxynitrite. In contrast, irisin mitigates endothelial cell dysfunction and delays the progression of atherosclerosis by inhibiting NOS-mediated oxidative and nitrative stress [93, 94]. Changes in plasma irisin levels were shown to correlate with circulating nitrite and nitrate levels, suggesting that increased irisin secretion leads to a reduction in arterial stiffness due to NO production [91]. The abovementioned antioxidant targets of irisin can contribute to the reversal of pathological processes induced by IRI.

Oxidative stress is one of the important mechanisms in IRI, whereas irisin can play an antioxidative stress role that can ameliorate IRI in distant organs. In terms of antioxidant systems, irisin protects the heart from IRI in a dose-dependent manner by means of increasing SOD activity and concentrations to decrease total ROS generation *in vivo* [95, 96]. In peri-infarct brain tissue, the rising levels of superoxide anion, nitrotyrosine, and 4-hydroxy-2-nonenals (4-HNEs) cause ischemia-induced neuronal injury, in which irisin exerts antioxidative and neuroprotective effects by activating protein kinase B (PKB) and extracellular-regulated protein kinase (ERK) pathways [97]. In intestinal IRI, irisin decreases levels of malondialdehydes (MDAs) and 4-HNE by activating GPXs, a process that benefits from the activation of the nuclear factor E2-related factor 2 (Nrf2) pathway [98]. In addition to the induced effect of irisin on the components of the antioxidant system (e.g., SOD, GPXs, and Nrf2), its inhibitory effect on oxidases (e.g., XO and NOX) has been observed in IRI-related diseases. Irisin does not only mediate the increased expression of SOD and GPXs, but also inhibits the XO system to synergistically enhance the antioxidant effect within intestinal tissues following intestinal IRI [99]. The IRI-induced heart infarct size can be reduced significantly by increasing levels of irisin and the NOX membrane subunit, gp91^phox^, encoding genes [100]. Interestingly, irisin-mediated negative regulation of NOX has been observed, suggesting that irisin might trigger a compensatory response to suppress an increase in NOX expression. In addition, iloprost and sildenafil improve ischemia-related tissue damage by increasing irisin concentrations and decreasing NO levels, but the correlation between irisin and NOS needs further clarification [94]. With regard to hemodynamic damage, irisin levels have been negatively correlated with the hemodynamic severity in pulmonary arterial hypertension [101]. Additionally, irisin can promote the proliferation of endothelial cells after flap revascularization to counteract reperfusion injury [102].

Overall, enzymic and nonenzymic oxidative stresses promote the generation of peroxide products (e.g., superoxide, H_2_O_2_, and ROS), causing an imbalance in oxidant-antioxidant systems *in vivo*. The accumulation of such products induces a direct toxicity in cells and tissues, in which XO, NOX, and NOS are primary oxidases related to IRI. Irisin exerts protective effects on IRI by activation of the antioxidant system and inhibition of the expression and activity of oxidases (see Table 1). However, little is known about the mechanisms that regulate irisin-mediated inhibition of IRI-related oxidative stress. Given that irisin regulates the three major oxidase systems and the antioxidant system, the following questions emerge: First, is the regulation of oxidant and antioxidant systems by irisin homogeneous in various tissues and affected by IRI? Second, what general features of these IRI-related disorders mediate their sensitivity to irisin regulation? Third, considering the relationship between NO, oxidases, and IRI, is the heterogeneous regulation for NO in IRI also mediated by irisin? Fourth, irisin might regulate oxidases and the components of the antioxidant system via multiple mechanisms. Are the protective effects of irisin and the existing therapies for IRI synergetic or redundant?

### 4.2. Mitochondrial Protection by Irisin during IRI

Irisin-mediated thermogenesis and energy expenditure are closely related to the biological functions of mitochondria, in which uncoupling proteins (UCPs) regulate mitochondrial functions within various tissues [95, 99, 103, 104]. Mitochondrial dysfunction plays a critical role during pulmonary IRI. The interaction between irisin and UCP2 inhibits decomposition of UCP2 after pulmonary IRI, reducing lung damage due to mitochondrial dysfunction derived-ROS [105]. Similarly, irisin is protective in hepatic and renal models of IRI through the upregulation of UCP2 [68, 106]. Decreased UCP2 levels result in direct damage to the mitochondrial DNA by ROS, leading to mitochondrial dysfunction and cell death. Ischemic postconditioning may exert mitochondrial protection by affecting the level of UCP3 and irisin during myocardial IRI [107]. Moreover, irisin plays a role in modifying the mitochondrial membrane potential (MMP) and mPTP openings. According to reports about MMP and mPTP, the rising ROS levels during IRI sensitize the opening of the mPTP, which causes the collapse of the MMP, leading to necrosis and tissue damage. To verify the targeting effect of irisin in mitochondria, Wang et al. [96] demonstrated that irisin restores the localization of SOD2 in mitochondria and interrupts ROS-mediated MMP collapse and mPTP opening in the heart following IRI. Under hypoxic and reoxygenating conditions, the curtailment of MMP and mPTP activity was apparent. Indeed, both collapse of the MMP and the opening of mPTP were rescued by irisin administration [95]. To verify the effect of irisin on mitochondria in further detail, Zhao et al. [108] found that exogenous irisin interacted with SOD2 within the mitochondria of cardiomyocytes. With respect to the intrinsic mechanism, this group demonstrated that irisin might rescue IRI-induced calcium overload in cardiomyocytes, which further impedes mPTP opening, thus, maintaining the integrity of the mitochondrial structure and function [95]. Moreover, irisin curtailed the mPTP opening by degrading histone deacetylase 4 to improve hypoxia and reoxygenation-mediated damage in cardiomyocytes [108].

Maintaining the balance of mitochondrial dynamics serves a pivotal role in cellular energy homeostasis. The continuous metabolic stress caused by hepatic IRI leads to a depletion of the intracellular ATP concentration, resulting in upregulation of Drp1, Fis1 expression, and increased mitochondrial fission in an effort to retain mitochondrial quantity and quality [109]. However, overactivated mitochondrial fission can give rise to mitochondrial fragmentation and apoptosis, thereby exacerbating tissue damage [110]. As a significant regulator that induces autophagy, irisin increases the expression of Opa1 which activates mitophagy, leading to scavenging of damaged mitochondria in cardiomyocytes which alleviates IRI after myocardial infarction [76, 111]. The literature suggests that irisin also improves myopathy caused by critical limb ischemia in aged mice by enhancing mitochondrial fission and mitophagy [112]. Meanwhile, the decline of PGC1-*α* expression, a vital regulator in mitochondrial biogenesis, and its downstream target mitochondrial transcription factor A (TFAM) both impair mitochondrial biogenesis under ischemia and hypoxia conditions, causing functional deterioration following cerebral IRI [113]. Both overactivated mitochondrial fission and failing mitochondrial biogenesis can be reversed by administering exogenous irisin which triggers the expression of mitochondrial fission-related proteins (e.g., Drp1 and Fis1) and mitochondrial biogenesis-related molecules (e.g., PGC1-*α* and TFAM), in turn protecting against hepatic IRI [68].

In summary, ETC-related mitochondrial oxidative stress, abnormal mitochondrial membrane potential and permeability, and unbalanced mitochondrial dynamics mediate mitochondrial dysfunction and contribute to irreversible cell death, thereby exacerbating IRI. Except for the antioxidant effects produced by the interplay between irisin and UCPs, irisin suppresses mitochondrial permeability by reducing mPTP opening. Irisin is also involved in regulating the expression of mitochondrial quality control-related molecules. All of abovementioned effects can significantly delay hypoxia or ischemia-induced mitochondrial dysfunction (see Table 1). Although numerous papers have been published about the effects of irisin on mitochondrial dysfunction during IRI, important questions remain: How does irisin regulate target molecules involved in IRI-induced mitochondrial dysfunction? Does irisin have an effect on reversing abnormal glucolipid metabolism in mitochondria caused by IRI? Does irisin protect against different types of cell death (e.g., apoptosis, necrosis, and ferroptosis) induced by mitochondrial dysfunction? Does excessive irisin trigger mitochondrial overdrive and noxious metabolite?

### 4.3. Anti-Inflammation of Irisin in IRI

The potential role of irisin as an anti-inflammation molecule has garnered widespread attention. The expression and activity of proinflammatory cytokines (e.g., TNF-*α* and interleukin-6) are enhanced as a result of obesity. They induce phosphorylation of inflammation-related nuclear factor kappa B (NF-*κ*B) and secrete proinflammatory “recruiting-related monocyte chemotactic protein 1” (MCP-1) [114]. Irisin suppresses the aforementioned proinflammatory response in a dose-dependent manner, while it also promotes phenotypic transformation of macrophages to synergistically exert an anti-inflammatory effect [114]. In a mouse model of atherosclerosis, irisin impedes the infiltration of inflammatory cells including T lymphocytes and macrophages into the atherosclerotic lesion [75]. Moreover, irisin upregulates the expression of microRNA126 to mitigate the adhesion of monocytes to the vascular endothelium [115]. Therefore, irisin inhibits the initial activation of the immune response under several pathological states. In addition, irisin blunts the activity of neutrophil elastase and fuels antioxidant function, contributing to the inhibition of the inflammatory cascade. Various studies have shown that irisin enhances the expression of Nrf2 in mice with chronic obstructive pulmonary disease (COPD). On one hand, Nrf2 balances the protease-antiprotease system in favor of suppressing COPD-induced emphysema. On the other hand, Nrf2 induces the expression of antioxidant enzymes and decreases the level of proinflammatory cytokines (e.g., interleukin-6 and interleukin-1*β*), which delays disease progression [116].

In view of its powerful anti-inflammatory effects, irisin may be considered for therapeutic strategies for IRI-related disorders. Exogenous irisin can relieve inflammation and liver injury in a rat model of hepatic IRI [78]. Inflammation caused by intestinal IRI could be blunted by irisin, and Nrf2 was shown to mediate such anti-inflammatory effects of irisin. The therapeutic potential of recombinant irisin in renal IRI has been demonstrated, since it works by reducing inflammation and tubular cell apoptosis [117]. Similarly, Kucuk et al. [118] showed that administration of irisin suppresses inflammation in a murine model of hind limb IRI evidenced by improvement of inflammatory biomarkers. Using oxygen-glucose deprivation (OGD) to mimic ischemic states *in vitro*, the expression of NOD-like receptor family, pyrin domain-containing 3 (NLRP3), and its downstream molecules (e.g., interleukin-1*β* and interleukin-18) were increased in a time-dependent manner. The administration of irisin reversed the activation of the NLRP3 inflammatory pathway induced by OGD, and as such exerted a neuroprotective effect in an ischemic stroke model [119]. Irisin can also stimulate the Notch signaling pathway to inhibit activity of microglial cells and inflammation, which also exerts a neuroprotective effect following cerebral IRI [120]. In addition, toll-like receptor-related pathways and NF-*κ*B activation induced mechanisms also play roles in protecting against neuronal injury and neurofunctional deficits after cerebral IRI [121].

As mentioned above, the activation of an initial inflammatory response and the subsequent amplification of inflammatory cascades during IRI can be ameliorated by irisin. Under ischemia and hypoxia, irisin inhibits the activation of proinflammatory pathways and the expression of inflammatory factors in order to protect against inflammatory injury following IRI (see Table 1). Current research mainly concentrates on verifying the anti-inflammatory effects of irisin during IRI, but the exploration of specific targets of irisin during the two inflammatory stages is lacking. Neutrophils are regarded as a central cell type for IRI-induced inflammation, but the effects of irisin on neutrophil haptotaxis, protease release, and oxidative burst during IRI remains unclear. In addition, the upregulated expression of antioxidation response element (e.g., Nrf2) mediated by irisin has an effect on suppressing the expression of proinflammatory genes in hypoxic disease as well as in IRI [98, 116]. Consequently, it is reasonable to study both the antioxidant and anti-inflammatory effects of Nrf2 and how they contribute to the regulation of multiple targets by irisin.

### 4.4. The Regulatory Signaling between Irisin and IRI

The expression of mitochondrial ubiquitin ligase MITOL is upregulated after administration of irisin, and it can exert a cardioprotective effect against myocardial IRI. MITOL is a vital molecule that regulates the interaction between the endoplasmic reticulum (ER) and mitochondria. During myocardial IRI, irisin increases the expression of MITOL, which in turn causes the “inositol-requiring kinase enzyme 1*α*” (IRE1*α*), a significant sensor in ER stress, to be ubiquitylated by MITOL [122]. Such an anti-ER stress effect of irisin further exerts cardioprotection via inhibition of the expression of apoptosis-related proteins. Moreover, upregulated MITOL exerts antioxidative stress effects and regulates mitochondrial dynamics following myocardial IRI. With respect to the anti-ER stress effect of irisin, there have been reports that irisin inhibits ER stress and oxidative stress by upregulating the level of GPX4, a phospholipid hydroperoxidase that protects against cell membrane lipid peroxidation. Increased GPX4 levels protect against ischemia-reperfusion-induced acute kidney injury [123]. ER stress and mitochondrial dysfunction also play significant roles in severe acute pancreatitis, where both effects can be suppressed by irisin [124]. Moreover, exogenous irisin inhibits ER stress and oxidative stress, and it exerts protection against mitochondrial dysfunction by improving the intestinal epithelial barrier function following intestinal IRI though the integrin *α*V/*β*5-AMPK-UCP 2 pathway [99]. Kindlin-2 can interact with the irisin receptor integrin *α*V/*β*5, and this interaction improves oxidative stress and mitochondrial dysfunction and inhibits ER stress and inflammation, in a mouse model of nonalcoholic fatty liver disease [125].

The adenosine 5′-monophosphate-activated protein kinase (AMPK) signaling pathway is another key hub for connecting irisin and IRI. In diabetic FNDC5 knockout mice subjected to myocardial IRI, cardiac function was found to have deteriorated, and the ischemic area was enlarged due to impaired AMPK signaling. In terms of the mechanisms, irisin promotes AMPK signaling to improve glucose uptake and glycolysis during ischemia; furthermore, the activity of AMPK signaling triggered by irisin alleviates mitochondrial dysfunction [126]. In addition, AMPK promotes deacetylation of PGC1-*α* and then increases the expression of irisin, which relieves oxidative stress during cardiac IRI (e.g., cardiorenal syndrome). Notably, aerobic exercise or genetic knockout of lysocardiolipin acyltransferase 1 (ALCAT1) activates the aforementioned AMPK-PGC1-*α*-irisin signaling cascade, thereby alleviating the oxidative stress-triggered mitochondrial dysfunction and cell apoptosis in kidneys of mice with myocardial infarction [127].

Ubiquitination and deacetylation are also involved in the protective effects of irisin for IRI. Small ubiquitin-like modification (SUMO) participates in rescuing hypoxia/reoxygenation-mediated injury after administration of irisin [108]. Irisin can induce sumoylation of histone deacetylases (HDAC), mitigating HDAC4-induced mitochondrial dysfunction during hypoxia/reoxygenation-mediated injury [108]. At the translational level, irisin promotes the upregulation of brain-derived neurotrophic factor (BDNF) and downregulation of matrix metalloproteinase-9 (MMP9), both of which exert a neuroprotective effect in cerebral IRI [128]. Furthermore, irisin has proangiogenic effects during heart IRI as a result of activating ERK pathway [129]. Finally, irisin-mediated inactivation of tumor suppressor gene P53 also curtails inflammation following renal IRI [117].

The regulatory signaling pathways mentioned above mediate the effects of irisin during IRI in various aspects and provide clues for new ways to supplement IRI therapeutic strategies (see Table 1). Irisin seems to be a pharmacologically tractable node for counteracting IRI, and the role of irisin in modulating intracellular metabolism under different stresses has garnered widespread attention. To date, studies on the therapeutic effects of irisin during IRI remains scarce, and little is known about the regulation of irisin at the transcriptional level. For example, nothing is known yet about the potential regulation of irisin by noncoding RNA [130]. The exact mechanisms responsible for the effects of irisin on IRI are far from being completely understood. The different roles of the same molecule in various pathways require further investigation, so that the full extent of irisin treatment against IRI-related diseases can be understood at the mechanistic level.

## 5. Discussion and Future Directions

**Figure 3:**
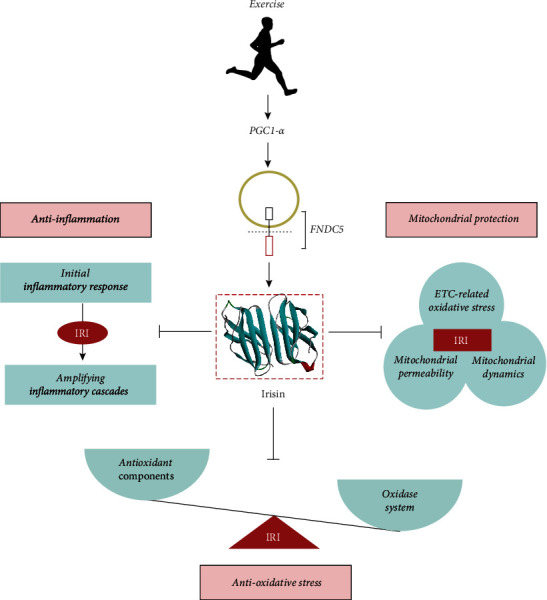
The mechanistic correlation between irisin and IRI. Exercise induces the expression of transcriptional coactivator PGC1-*α* and its downstream gene FNDC5, after which FNDC5 is cleaved and secreted into the circulation. An effective splice product of FNDC5 is named irisin. (1) Imbalance of the oxidant-antioxidant system triggers oxidative stress during IRI. Irisin exerts protective effects on IRI by activating the antioxidant components and inhibiting the expression and activity of oxidases. (2) ETC-related mitochondrial oxidative stress, abnormal mitochondrial permeability, and unbalanced mitochondrial dynamics all mediate mitochondrial dysfunction and IRI deterioration, while irisin exerts mitochondrial protection to reverse above changes. (3) The activation of the initial inflammatory response and the subsequent amplification of the inflammatory cascades during IRI can be improved by irisin. ETC: electron transport chain. FNDC5: fibronectin type III domain-containing protein 5; IRI: ischemia-reperfusion injury; PGC1-*α*: PPAR-c coactivator-1 *α*.

Ischemia-reperfusion injury (IRI) is a central feature of various disease processes, and it can be a primary cause for high morbidity and case fatality rates. Oxidative stress, mitochondrial dysfunction, and an inflammatory response are crucial elements of the pathogenesis of IRI. Irisin is a myokine that was discovered in 2012, which is produced by cleavage of the c-terminus of FNDC5 [65]. The antioxidant and anti-inflammatory effects as well as the mitochondrial protective effects of irisin have been demonstrated in various studies. These effects of irisin overlap with the pathogenic mechanisms of IRI (see Figure 3) In this review article, we first focused on the pathogenesis of IRI, dissecting the inherent mechanisms of IRI including oxidative stress, mitochondrial dysfunction, and the inflammatory response, as well as the main molecules involved in those pathways. Next, we summarized how irisin modulates IRI through its effects on oxidative stress, mitochondrial dysfunction, and inflammation. The purpose of this review was also to identify gaps in knowledge and to provide ideas for new research directions aimed at developing new therapeutic strategies with precision and multiple targets for the treatment of IRI. Future studies into the mechanistic effects of irisin can enable translational studies that can potentially be extended into clinical applications.

There are some gaps in our knowledge of irisin that need to be studied: (1) The effective targets and regulatory pathways are yet to be ascertained. (2) There is a gap between fundamental research studies and clinical studies in this area. (3) The technical difficulties in detecting irisin needs to be improved. Nevertheless, it appears that irisin has a great therapeutic potential based on IRI-related studies in recent years. Firstly, an abnormal concentration of irisin in plasma is regarded as a candidate biomarker for organ damage under pathological conditions (e.g., cardiovascular disease, metabolic disease, and tumor) [87, 131–133]. In light of this, we postulate that irisin could serve as a biomarker for IRI, for the diagnosis for organ damage, for the assessment of the severity of the injury, or for the potential protective effect of a treatment. Second, it is unclear whether the administration of exogenous irisin will cooperate with existing treatments for IRI-related disorders. Moreover, it would be important to assess the potential benefits of a combined treatment involving irisin and remote ischemic preconditioning. In conclusion, the current literature suggests that irisin holds great promise for the treatment if IRI. Technological improvements and extensive experimental and clinical research are needed to accelerate the potential development of an irisin-based treatment for IRI-related disorders.

## Figures and Tables

**Table 1:** The effects and mechanisms of irisin during ischemia-reperfusion injury.

Organs	Effects on IRI	Mechanisms involving irisin	Ref.
Cerebral IRI	Inflammation↓	NLRP3 pathway	[119]
	Inflammation↓	Notch pathway	[120, 121]
	O_2_^•-^↓, MDA↓, 4-HNEs↓	PKB and ERK pathways	[97]
	PGC1-*α*↑, TFAM↑	Mitochondrial biogenesis	[113]
Flap revascularization	Endothelial cell proliferation	Antioxidative stress	[102]
Heart IRI	HDAC4 degradation↓	HDAC sumoylation	[108]
	MITOL↑	Anti-ER stress	[122]
	NOX↓	Antioxidative stress	[100]
	Opa1↑	Mitochondrial fusion	[42]
	Opa1↑	Mitophagy	[76, 111]
	PGC1-*α* deacetylation	AMPK pathway	[126, 127]
	Proangiogenic function	ERK pathway	[129]
	ROS↓, SOD↑	Antioxidative stress	[95, 96]
	SOD2↑, calcium overload↓	Mitochondrial permeability	[95]
	SOD2 localization	Mitochondrial permeability	[96]
	UCP3↑	Anti-mtROS	[107]
Hepatic IRI	Drp1↓, Fis1↓	Mitochondrial fission	[68]
	PGC1-*α*↑, TFAM↑	Mitochondrial biogenesis	[68]
	Kindlin-2↑	Anti-ER stress	[125]
	UCP2↑	Anti-mtROS	[68]
Hind limb IRI	Inflammatory biomarkers↓	Anti-inflammation	[118]
Intestinal IRI	Inflammation↓	Anti-ER stress	[99]
	MDAs↓, 4-HNEs↓, GPXs↑	Antioxidative stress	[98]
	SOD↑, GPXs↑, XO↓	Antioxidative stress	[99]
Pulmonary IRI	UCP2↑	Anti-mtROS	[105]
Renal IRI	Inflammation↓	Anti-ER stress	[117]
	Inflammation↓	P53 inactivation	[117]
	UCP2↑	Anti-mtROS	[106]

AMPK: adenosine 5′-monophosphate-activated protein kinase; Drp1: dynamin-related protein 1; ERK: extracellular-regulated protein kinases; ER stress: endoplasmic reticulum stress; Fis1: fission protein 1; GPXs: glutathione peroxidases; HDAC: histone deacetylases; 4-HNEs: 4-hydroxy-2-nonenals; IRI: ischemia-reperfusion injury; MDAs: malondialdehydes; MMP: mitochondrial membrane potential; mPTP: mitochondrial permeability transition pore; mtROS: mitochondrial reactive oxygen species; NOX: NADPH oxidase; O_2_^•-^: superoxide; Opa 1: optic atrophy 1; PGC1-*α*: PPAR-c coactivator-1*α*; PKB: protein kinase B; SOD: superoxide dismutase; TFAM: target mitochondrial transcription factor A; UCPs: uncoupling proteins; XO: xanthine oxidase; ↑: increase; ↓: decrease; /: “or”.

## Abbreviations

ALCAT1: Aerobic exercise or genetic knockout of lysocardiolipin acyltransferase 1
AMPK: Adenosine 5′-monophosphate-activated protein kinase
ATP: Adenosine triphosphate
BDNF: Brain-derived neurotrophic factor
BH_4_: Tetrahydrobiopterin
CAD: Coronary artery disease
COPD: Chronic obstructive pulmonary disease
CoQ: Coenzyme Q
DAMPs: Damage-associated molecular patterns
Drp1: Dynamin-related protein 1
ER: Endoplasmic reticulum
ERK: Extracellular-regulated protein kinases
ETC: Electron transport chain
FAD: Flavin adenine dinucleotide
Fis1: Fission protein 1
FMN: Flavin mononucleotide
FNDC5: Fibronectin type III domain-containing protein 5
GPXs: Glutathione peroxidases
HDAC: Histone deacetylases
4-HNEs: 4-Hydroxy-2-nonenals
H_2_O_2_: Hydrogen peroxide
HIF-1*α*: Hypoxia-inducible factor-1*α*
HOCl: Hypochlorous acid
IRE1*α*: Inositol-requiring kinase enzyme 1*α*
IRI: Ischemia-reperfusion injury
L-Arg: L-arginine
L-Cit: L-citrulline
MCP-1: Monocyte chemotactic protein 1
MDAs: Malondialdehydes
MMP: Mitochondrial membrane potential
MMP9: Matrix metalloproteinase-9
mPTP: Mitochondrial permeability transition pore
mtROS: Mitochondrial ROS
NADPH: Nicotinamide adenine dinucleotide phosphate
NF-*κ*B: Nuclear factor kappa B
NLRP3: NOD-like receptor family, pyrin domain-containing 3
NO: Nitric oxide
NOS: Nitric oxide synthase
NOX: NADPH oxidase
Nrf2: Nuclear factor E2-related factor 2
O_2_: Oxygen
O_2_^•-^: Superoxide
OCl^−^: Hypochlorite
OGD: Oxygen-glucose deprivation
^•^OH: Hydroxyl radical
Opa1: Optic atrophy 1
PGC1-*α*: PPAR-c coactivator-1*α*
PKB: Protein kinase B
PPAR: Peroxisome proliferator-activated receptor
RET: Reverse electron transport
ROS: Reactive oxygen
SCD: Sickle cell disease
SOD1: Superoxide dismutase 1
sRBC: Sickle red blood cells
SUMO: Small ubiquitin-like modification
TFAM: Mitochondrial transcription factor A
TNF-*α*: Tumor necrosis factor *α*
UCPs: Uncoupling proteins
XD: Xanthine dehydrogenase
XO: Xanthine oxidase.
